# Ecotoxicological Assessment of Sediment Samples Impacted by Wastewater Treatment Plant Effluents Transporting Contaminants of Emerging Concern

**DOI:** 10.3390/jox15040132

**Published:** 2025-08-15

**Authors:** Carlos Silva, Ana Ré, Nelson Abrantes, Fernando J. M. Gonçalves, Joana Luísa Pereira

**Affiliations:** 1 CESAM—Centre for Environmental and Marine Studies and Department of Biology, University of Aveiro, 3810-193 Aveiro, Portugal; carlos.dias.silva@ua.pt (C.S.); njabrantes@ua.pt (N.A.); fjmg@ua.pt (F.J.M.G.); 2 CESAM—Centre for Environmental and Marine Studies and Department of Environment and Planning, University of Aveiro, 3810-193 Aveiro, Portugal; anare@ua.pt

**Keywords:** toxicity testing, test battery, sediment testing, pharmaceuticals, river contamination

## Abstract

Wastewater treatment plant (WWTP) effluents can be important sources of contaminants of emerging concern (CEC) for riverine ecosystems, with some accumulation in sediments. This study investigated the ecotoxicological effects of sediment samples collected near three WWTPs. Sediment elutriates, simulating resuspension conditions, and whole sediment samples were tested. Results showed that sediments were toxic to some organisms and beneficial to others. Elutriates from one site significantly reduced luminescence in the bacterium *Aliivibrio fischeri*, though this was not consistently linked to sediment contaminant levels. Significant noxious effects of elutriates were recorded for the macrophyte *Lemma minor* (yield reductions up to 48%) and the microalgae *Raphidocelis subcapitata* (yield reductions up to 25%). Exposure to elutriates resulted in increased *Daphnia magna* reproduction and increased biomass yield of *Chironomus riparius* exposed to sediments directly. Overall, there were no major toxicity variations in samples collected upstream and downstream of the effluent outfall. Suggesting limited hazardous potential of the effluent and a potential masking effect of background contamination (mostly metals and polycyclic aromatic hydrocarbons). The complexity of effluent-sourced contamination, coupled with the realistic testing approach, renders this work a valuable contribution to understanding the role of WWTP effluents in surface freshwaters contamination and their effects, especially concerning CECs.

## 1. Introduction

Conventional wastewater treatment plants (WWTPs) were not specifically designed to efficiently remove some of the chemicals they now receive, making it common that some are still detected in treated effluents [1,2]. Among those frequently present in treated effluents are the so-called contaminants of emerging concern (CEC), a wide group of chemicals that were either recently detected in the environment and/or recently recognized as a potential threat to human or ecosystem health [3]. It is common to find tens of different CECs, such as pharmaceuticals, industrial chemicals, pesticides, in treated effluents [4,5,6], and although they are usually detected at low concentrations there is evidence of effects of exposure to effluents containing them. Exposure to treated WWTP effluents have been linked to alterations of macroinvertebrate abundance and community composition [7], increased incidence and severity of intersex [8] and increased antioxidant activity [9] in fish, among other effects.

For most CECs, there are knowledge gaps regarding potential effects in the environment, and some, such as pharmaceuticals, were designed to be bioactive, increasing the likelihood of impairing non-target biota [10]. Worsening the potentially hazardous scenario, treated WWTP effluents frequently include a complex mixture of CECs and legacy chemicals, often incorporating metals [11], polycyclic aromatic hydrocarbons (PAHs), pharmaceuticals and personal care products (PPCP) [12] biocides [13], among others. This complexity makes toxic effects difficult to predict due to interactions that may occur between the components of the mixture rendering the effects non-additive [14]. Therefore, assessing environmental matrices affected by effluents retrospectively is a feasible approach to understand their hazardous potential.

Effluents treated by inland WWTPs are typically discharged in waterways, which are often natural or minimally modified. In Europe, there are requirements for effluent discharges, but these are mainly focused on biochemical parameters (oxygen demand, total suspended solids, total phosphorous and total nitrogen) and on a list of priority substances (defined by directive 2013/39/EU). Most chemicals, including numerous CECs, are not regulated, and key characteristics of the receptor media, such as dilution capacity or flow speed, are often insufficiently considered. Furthermore, although the water framework directive (WFD) assessment tools are comprehensive, incorporating both chemical and biological indicators to address the quality of water bodies, there is limited integration between this directive and effluent discharge regulations, which could enhance management strategies. Moreover, the WFD’s bioassessment protocols are technically complex and time-consuming, posing challenges for WWTPs to align with their effluent quality monitoring schedules. In this context, ecotoxicological testing emerges as a promising alternative, providing a more direct link between cause and effect and potentially enabling the adjustment of effluent standards to align with specific ecosystem characteristics [15].

In riverine ecosystems affected by WWTP effluents, as in any lotic freshwater ecosystem, the sediment and the benthos assume particular ecological relevance since adverse scenarios that may occur in the water column (e.g., discharge of a contaminated effluent) are transient due to the flow. Sediments are important sinks, and potential sources of contaminants in cases where remobilization occurs [16], in freshwater ecosystems and especially in lotic systems. Over time, the accumulation of contaminants in sediments gives an historical perspective that water column analysis cannot capture due to the continuous flow. This accumulation also results in chronic exposure of benthic organisms to contaminants, potentially at increasing concentrations. Altogether, these features make sediment a crucial environmental matrix for assessing the environmental hazardous potential of treated WWTP effluents discharges.

Ecotoxicological testing with sediment can follow different approaches, with potentially different outcomes [17], including the use of whole sediment or elutriates. Sediment elutriate toxicity tests were designed to evaluate the impact of contaminants transferred to the water phase due to sediment resuspension, while whole sediment test evaluates toxicity directly, integrating different exposure routes. Furthermore, differences in toxicity levels within each type of test can be found depending on the organisms used, as different species exhibit varying sensitivities to contaminants due to distinct uptake routes and metabolic pathways. This highlights the importance of using multiple organisms in toxicity evaluations to avoid over-or underestimating the hazardous potential. This approach is especially critical when assessing field samples, where full knowledge of sample composition is often unknown to support a restrictive selection of test organisms.

Under the reasoning above, in the present study, we assessed the ecotoxicity of sediments collected in streams affected by the discharge of treated WWTP effluents. For that, we employed a battery of standard assays using well-established model organisms and sub-lethal endpoints. Elutriates were tested with the Microtox^®^ assay, assessing the inhibition of luminesce by the bacterium *Aliivibrio fischeri*; the growth inhibition test with the microalgae *Raphidocelis subcapitata*; the growth inhibition test with the aquatic plant *Lemna minor*; the reproduction test with the crustacean zooplankter *Daphnia magna*. Whole sediment was tested to assess the growth of larvae of the dipteran *Chironomus riparius*. Sediment samples were collected in the vicinity of three WWTPs. In each one, one sample was collected upstream the effluent outfall, another immediately downstream and a third further downstream the effluent outfall, the latter covering for differential flow patterns that impact the transport and sinking of contaminants. We hypothesized that (i) the contaminants present in the sediment cause measurable spatial differences in the evaluated endpoints across the test organisms, highlighting the hazard potential of the sediments; and that (ii) samples collected upstream and downstream of the effluent outfall induce different toxicity outcomes, shedding light on a potential causal relationship with the WWTP effluent discharge.

## 2. Materials and Methods

### 2.1. Sediment Sampling and Sample Preparation for Testing

Three Portuguese wastewater treatment plants (WWTP) were selected as case studies (WWTPa WWTPb and WWTPc) each differing in treatment methods, population equivalents served, as well as on the contamination patterns in their effluents and in the sediments of the recipient waterways. Those aspects were thoroughly explored in Silva et al. [18] and are summarized in Table S1. For each WWTP case study, we selected three sampling sites in the discharge waterway: one immediately upstream the effluent outfall (Upa, Upb, Upc); a second downstream (D1a, D1b, D1c); and a third located at least 500 m further downstream (D2a, D2b, D2c). Comparison between Up and D1 samples allows isolating the effluent as a contamination source; although still under the potential influence of the effluent, D2 samples allow insights into the dilution of its hazardous effects depending for example on flow patterns, water properties and riverbed sediment transport. At each sampling site, a composite sediment sample (surface layer; top 20 cm) was collected with a shovel by pooling from at least 5 sub-sites to account for riverbed heterogeneity. Samples were transported to the laboratory in an ice cooler and briefly stored at −20 °C until testing. Immediately before testing with bacteria, microalgae, macrophytes and daphnids (see Section 2.2), elutriates were prepared in each test medium (see Section 2.2 for the media used for testing with each organism) using a 1:4 sediment to test medium proportion [19]. The mixture was left shaking overnight on an orbital shaker at 200 rpm at 20 ± 2 °C; then, it was centrifuged at 2000× *g* for 20 min, decanted, and the supernatant was stored at 4 °C until use.

### 2.2. Test Organisms and Test Setup

#### 2.2.1. *Aliivibrio fischeri* (Microtox^®^Test)

*Aliivibrio fischeri* stock vials and test reagents were bought from Modern Water Inc. (New Castle, DE, USA), and samples were tested under the Microtox^®^ 81.9% basic test protocol according to the manufacturer recommendations. The Microtox^®^ test kit (Modern Water Inc., New Castle, DE, USA) is based on the fact that exposure to toxic substances disrupts the bacteria’s respiratory processes resulting in a reduction in luminescence [20]. Briefly, the bacteria were exposed to a series of dilutions (0%; 0.32%; 0.64%; 1.28%; 2.56%; 5.12%; 10.24%; 20.48%; 40.95%; 81.90%) of each test sediment elutriate, and differences in luminescence compared to an uncontaminated control were measured (30 min after exposure) using a Modern Water Inc. model 500 analyser (New Castle, DE, USA).

#### 2.2.2. *Lemna minor*

*Lemna minor* was cultured in Steinberg medium [21] at 20 ± 2 °C with a 16 h L:8 h D photoperiod with scheduled culture renewals once a week. Growth inhibition tests followed the recommendation of the OECD guideline 221 [22]. Tests were carried out in 6-well microplates, with 10 mL test solution per well (increasingly diluted sediment elutriate in Steinberg or blank Steinberg for controls), as adapted by Kaza et al. [23] and Kolasińska et al. [24], at 23 ± 1 °C, under continuous light. Each replicate well was inoculated with 3 healthy colonies of 3 fronds each at the beginning of the test. Each treatment was tested in triplicate with 6 replicates assigned to the control treatment. After 7 days of exposure, all fronds in each plate were collected and counted. The impacts in *L. minor* growth were assessed based on changes in frond number yield through elutriate treatments.

#### 2.2.3. *Raphidocelis subcapitata*

The green microalga *R. subcapitata* was cultured in sterilized Woods Hole MBL medium [25] at 20 ± 2 °C with a 16 h L:8 h D photoperiod with scheduled renewals once a week. Tests followed the OECD guidelines No. 201 [26] adapted for testing with reduced volumes [27] in 24-well microplates containing 1 mL of test solution (increasingly diluted sediment elutriate in MBL or blank MBL for controls) per well, using an initial cell density of 10^4^ cells/mL adjusted after microscopic counting under a Neubauer hemocytometer. Incubation lasted for 96 h, at 23 ± 1 °C under continuous light. To prevent cell clumping and promote gas exchange the algal suspension in each well was mixed by repetitive pipetting twice a day. At the end of the test, optical density was read (440 nm; Shimadzu UV−1800; Kyoto, Japan) in each well and converted to cell density records using a previously established calibration curve. Each treatment was tested in triplicate. Growth was assessed based on the biomass yield as shown by cell density.

#### 2.2.4. *Daphnia magna*

*Daphnia magna* monoclonal cultures (Beak clone) were reared in ASTM hard water medium [28] enriched with vitamins and supplemented with an organic additive (*Ascophyllum nodosum* extract) [29], kept at 20 ± 2 °C, under a photoperiod of 16:8 h light:dark. Culture media was changed 3 times per week, and animals concomitantly fed with *R. subcapitata* (3.0 × 10^5^ cells mL^−1^) cultured as described in Section 2.2.3. *Daphnia magna* neonates, ageing less than 24 h and born between the 3rd and 5th brood were used for tests. Testing was done following the OECD guideline 211 [30]. Neonates (10 for each treatment) were exposed for 21 days in individual 100 mL vials, with 10 mL test solution. Renewal of whole test solutions and feeding following the same scheme described above for bulk cultures; organic supplementation was not made as recommended by the guidelines and considering the expected presence in the samples of chemicals that absorb to organic matter (see [18]). In this test, no dilutions of the elutriate treatment were tested; dilutions would be tested in a follow-up test stage provided the confirmation of negative effects induced by the full-strength elutriate. The reproductive output was recorded daily and derived endpoints (age at first reproduction, size of the first brood and total number of neonates yielded per female) were calculated.

#### 2.2.5. *Chironomus riparius*

Midge egg ropes were obtained from an in-house laboratory culture, maintained in crystallized dishes, with inorganic <1 mm river sand (burnt at 450 °C during 4 h) and soft water prepared according to the OECD guideline 233 [31]. Cultures were maintained at 20 ± 1 °C, under a photoperiod of 16:8 h light/dark, and constant aeration. The medium was renewed twice a week, and the larvae were fed ad libitum with macerated fish food flakes (Tetramin^®^, Melle, Germany). Tests were started with five recently hatched larvae (<24 h) placed in 600-mL (ø = 11 cm) crystallizing dishes, each containing a 1.5 cm bottom layer of sediment sample or the sand used for cultures in the case of the control and under constant aeration–no renewal of the setup was carried out during the test. Each treatment (non-diluted sediment sample) had 5 replicates. Larvae were fed macerated Tetramin^®^ at a rate of 0.25 mg larvae^−1^ day^−1^ throughout the experiment (10 days). At the end of the experiment, larvae were collected from sediment, counted and dried at 60 °C for 24 h and individual dry weight measurement. Apart from manually cleaning of larger detritus, sediment samples were used as collected. Water quality parameters (temperature, pH, dissolved oxygen and conductivity) were monitored through the test, confirming compliance with the guideline recommendations.

#### 2.2.6. Statistical Analysis

One-way ANOVA, followed by a Dunnett post hoc test was used to assess the effects of elutriate dilution within each site and each WWTP on the endpoints assessed with *L. minor* and *R. subcapitata*. For treatments where significant differences with the test control were found (regardless of the site where these differences were noticed), a second one-way ANOVA followed by a Tukey post hoc test was performed to assess differences among sites. Concerning the endpoints assessed with *D. magna* and *C. riparius* (where elutriate or sediment was not diluted for testing), a one-way ANOVA followed by the Tukey post hoc test was used to evaluate the effect of the site within each WWTP. When data failed ANOVA assumptions of normality and homoscedasticity (verified via the Shapiro–Wilk and Levene tests, respectively), non-parametric equivalents were rather used: the Kruskal–Wallis test followed by a Dunn’s post hoc test to assign significant differences among groups.

## 3. Results and Discussion

### 3.1. Effects of Elutriates in Aliivibrio fischeri Luminescence

**Figure 1:**
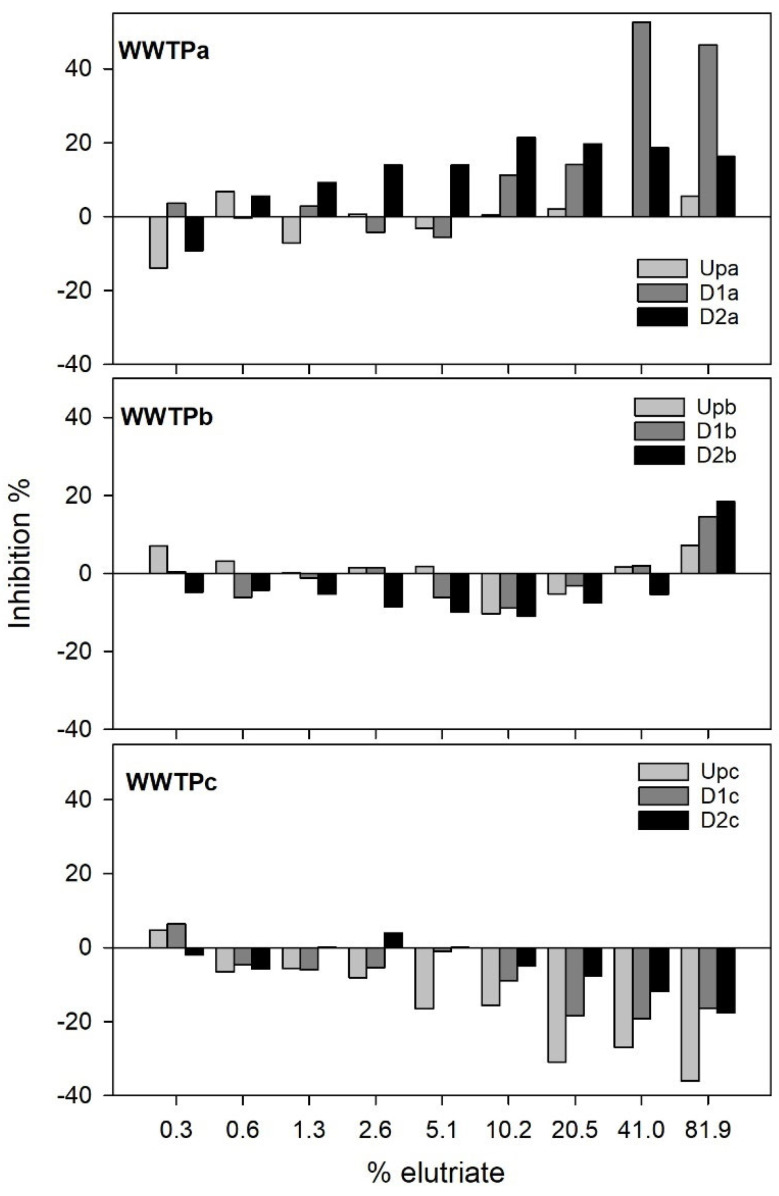
Percent inhibition in *Aliivibrio fischeri* luminescence after exposure to different elutriate concentrations (%). Inhibition was calculated by comparing light emitted by a control sample and that emitted in each of the elutriate dilutions.

Figure 1 shows *A. fischeri* bioluminescence inhibition promoted by the sediment samples after 30 min of exposure to sediment elutriates.

Exposure to sediment elutriates impacted bioluminescence, but this impact was clearly negative only for samples collected in WWTPa, while for the other WWTP, bioluminescence was basically stimulated by the sediment elutriate samples, with the exception of the highest concentration of WWTPb elutriates. Sediment samples from WWTPa consistently induced increased inhibition for the highest concentrations of elutriates tested, and higher toxicity was found for sites downstream of the effluent outfall, particularly in D1a samples. For WWTPb only the highest concentration of elutriate (89.1%) resulted in mild percentages of luminescence inhibition (not surpassing 20% regardless of the site), with the D2b sample inducing slightly higher inhibition when compared with the other two samples. *Proteobacteria*, including *A. fischeri* are generally considered particularly sensitive to antibiotics [32], which could contribute to explain the significant luminescence inhibition observed after exposure to certain elutriate samples sourced by WWTPa and WWTPb. However, potentially conflicting evidence arises from the chemical analysis of these sediments [18]; Table S1. First, no PPCPs were found in sediment samples from WWTPa. Second, only one PPCP (trimethoprim) was quantified in WWTPb sediment samples, which was found in Upb and D1b at very low concentrations, ranging within 0.07–48 µg/kg.

Effluents are among the most complex mixtures of chemicals to which ecosystems are frequently exposed [14]. Although Silva et al. [18] conducted an extensive chemical characterization of sediment samples, the possibility that there were non-surveyed contaminants contributing to the observed PPCPs in samples from downstream the effluent outfall in WWTPa and WWTPb should not be ruled out. Furthermore, and considering that several antibiotics were quantified in the corresponding effluents [18], it is plausible that sediments still carry antibiotic residues, albeit below quantification limits. Multiple contaminants at low concentrations could theoretically still induce effects, if synergism is in place [33,34]. Conversely, despite the higher load of PPCPs found in WWTPc, no inhibition of bacterial luminescence was observed (see below), suggesting that the inhibitory effects found in WWTPa and WWTPb may be attributed to other types of contaminants. Metals are unlikely to be responsible for this inhibition, as the literature reports that even highly metal-contaminated sediment extracts (several orders of magnitude above the concentrations in our samples) tend to induce little or no bioluminescence inhibition [35,36]. This low toxicity of metal-contaminated sediments may be due to the limited mobilization of metals into the dissolved phase in elutriates, as shown by Hagner et al. [36] and Vidic et al. [37]. Similarly, Bispo et al. [38] found low levels of toxicity for *A. fischeri* in samples with PAH levels orders of magnitude higher than those reported in our samples. Therefore, it seems unlikely that metals or PAHs are responsible for the observed impacts on bioluminescence in WWTPa and WWTPb samples, whereas it is more likely that this effect resulted from either the combined action of various contaminants or the presence of unmonitored contaminants.

Regardless of the external stimuli in place, the inhibition of *A. fischeri* bioluminescence reflects disruption in cellular respiration and energy production, thus bacterial metabolic activity [39]; it can hence be interpreted as proxy for impairment in fundamental roles that bacteria play in sediments, such as organic matter and nutrient cycling. If taking *A. fischeri* as an indicator, our results suggest that microbial metabolic activity can be markedly compromised by the WWTPa effluent discharge, potentially impairing these key ecosystem functions; this link was already shown in different studies, for example regarding pesticide contamination and organic matter decomposition [40]. Furthermore, negative effects in microbial communities can surely cascade upwards in the food web, compromising resources availability at higher trophic levels (e.g., benthic macroinvertebrates) and linked ecosystem functions [41,42]. In WWTPc samples, a consistent stimulatory effect was noticed as elutriate concentrations increased, with the Upc sediments causing the highest stimulation levels compared to samples collected downstream of the effluent outfall (Figure 1). These stimulatory effects are not uncommon; for example, Davoren et al. [43] reported bio-stimulation in the Microtox^®^ assay after exposure to estuary sediment elutriates. Notably, sediments from WWTPc presented elevated concentrations of nutrients such as C (based on the organic matter), N, P and essential nutrients (Table S1) when compared to those from WWTPa and WWTPb. These nutrients are known to support microbial metabolism and can enhance *A. fischeri* bioluminescence through increased energy availability and cellular activity, particularly under the nutrient-poor assay conditions of the Microtox^®^ system. Provided that the Upc elutriate induced the highest stimulation, the stimulatory effect should have not been driven by the effluent and its contaminant burden. Silva et al. [18] found several PPCP in downstream WWTPc sediments, whereas only caffeine was detected in Upc (Table S1). The higher burden in several PPCPs of the samples downstream the effluent outfall may contribute to explain the lower stimulation in bioluminescence compared to Upc. This was also the site where sediments have the lowest metal loading, with generally higher concentrations downstream the effluent outfall (Table S1); but the opposite is interpretable regarding PAHs. Together, these findings suggest that the stimulatory effect observed at Upc is likely driven by a combination of nutrient availability and/or sub chronic effects caused by low levels of biologically active compounds, such as endocrine disruptors not detected in our analysis.

### 3.2. Effects of Elutriates in Autotrophs

**Figure 2:**
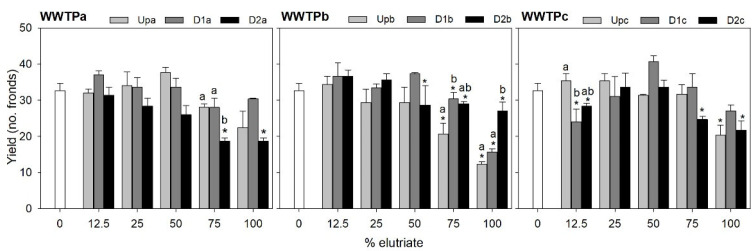
*Lemna minor* yield based on the number of fronds counted at the beginning and the end of the test. The bars represent the average of three replicates and the error bars represent the standard error. Asterisks denote the treatments (elutriate dilutions) significantly differing from the control at each site (Up, D1 or D2) within each WWTP (Dunnett test or the non-parametric equivalent Dunn’s; *p* < 0.05). Letters denote differences among sites within each treatment (Tukey test; *p* < 0.05) where differences compared to control for a site had been found previously in that treatment.

**Figure 3:**
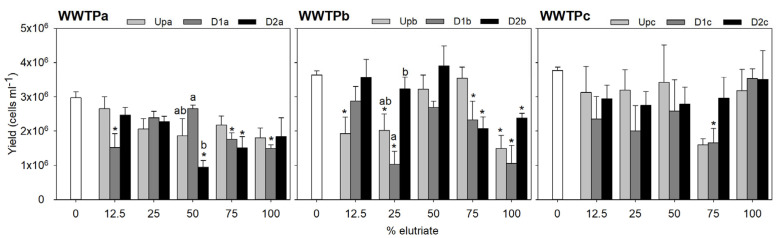
*Raphidocelis subcapitata* yield based on cell density assessed at the beginning and the end of the test. The bars represent the average of three replicates and the error bars represent the standard error. Asterisks denote the treatments (elutriate dilutions) significantly differing from the control at each site (Up, D1 or D2) within each WWTP (Dunnett test or the non-parametric equivalent Dunn’s; *p* < 0.05). Letters denote differences among sites within each treatment (Tukey test; *p* < 0.05) where differences compared to control for a site had been found previously in that treatment.

Sediment elutriates from all sampling sites generally impaired the growth of the macrophyte *L. minor* (Figure 2) and the microalgae *R. subcapitata* (Figure 3), assessed, respectively, based on yield of frond number and cell density. The inhibitory effects were most pronounced when plants were exposed to the undiluted elutriate. At lower elutriate concentrations, a stimulatory effect on plant growth was often observed, and in some cases, an increase in microalgal yield was also noted.

Growth inhibition resulting from exposure of *L. minor* and *R. subcapitata* to WWTPa sediments was less pronounced at Upa compared to D1a and D2a (Figure 2 and Figure 3). D2a generally elicited a higher inhibition in the macrophyte growth than D1a (with up to 43% inhibition in treatments comprising 75% and 100% elutriate), being significant for the 75% elutriate treatment (Figure 2; Table S2). A similar trend was found for the microalgae, although D1a was comparatively more harmful: the D1a sample significantly impaired microalgae growth at elutriate strengths of 12.5% (potential technical issue), 75% (41% inhibition) and 100% (50% inhibition), while the D2a sample impaired significantly the microalgae growth at 50% (69% inhibition) and 75% (49% inhibition) elutriate strengths (Figure 3; Table S2). Although no significant inhibition in yield could be corroborated by the statistics for Upa elutriates, a decreasing trend in *L. minor* yield compared to the internal test control is evident at 75% and 100% elutriate concentrations (Figure 2; Table S2). Similarly, in *R. subcapitata*, a reduction in yield is noticeable starting from 25% elutriate concentration onwards. This is particularly consistent with the metal burden of sediments upstream the effluent outfall in WWTPa, suggesting sources other than the effluent for metal sediment contamination [18]; Table S1. Apart from the notable exceptions of phosphorous (a nutrient for autotrophs) and copper (an essential element), Upa sediments hold the highest element concentrations compared to D1a and D2a (Table S1). Provided that the elutriate samples from sites downstream the effluent outfall generally induced stronger toxic effects in both species, it is reasonable to interpret that metals are not the main drivers of these effects. PAHs do not seem responsible for the growth inhibition observed in *L. minor*, as the concentrations capable of inducing reduction on growth rate, as reported by Čvančarová et al. [44], are several orders of magnitude higher than those in our samples. Similarly, Clément et al. [45] found no impact on growth after exposure to sediment samples with total PAHs concentrations higher than ours. PAHs are also unlikely to be toxic for *R. subcapitata* at the concentrations present in our sediments, as the concentrations associated with toxicity in Baun et al. [46] are several orders of magnitude above the levels quantified herein.

Samples from WWTPb impaired yield only in the 75% elutriate treatment (22–47% inhibition, depending on the site) and in undiluted elutriate (30–68% inhibition, depending on the site), whereas the remaining elutriate dilutions were not harmful or induced mild growth stimulation (Figure 2). In WWTPb, it is worth noting that the sample inducing the stronger decreases in *L. minor* growth was that collected upstream the effluent outfall; this effect was significantly stronger than that noted after exposure to D1b and/or D2b in the 75% elutriate treatment and in the undiluted elutriate (Figure 2; Table S2). Although WWTPb samples promoted a variable response in the microalgae with the increase in elutriate strength, a similar trend to *L. minor* was observed in microalgae yield for the undiluted elutriate treatment (Figure 3; Table S2). A significant decrease in microalgae growth was observed after exposure to the undiluted elutriate regardless of the site (59%, 71% and 34% inhibition for Upb, D1b and D2b, respectively). Exposure to the 75% of elutriate treatment also led to significant growth inhibition at sites downstream the effluent outfall (36% and 43% inhibition for D1b and D2b); albeit no significant impairment of microalgae growth was noted after exposure to the 75% Upb elutriate, there were no significant differences in the response between sites. Overall, the evidence above suggests that contamination sources other than the effluent are more important drivers of toxicity than the effluent itself, and/or that the downstream sites can have a positive influence in the macrophyte growth slightly offsetting the noxious impact of contaminants. Nutrient enrichment has been shown to increase autotrophic biomass [47], and indeed, sediments downstream the effluent outfall in WWTPb tend to be richer in essential macro and microelements that can favour macrophytes growth (Table S1).

WWTPc samples showed a tendency for growth stimulation at elutriate concentrations between 12.5% and 50%, whereas a significant growth impairment was found for *L. minor* exposed to the 75% elutriate from D2c (35% inhibition) and the 100% elutriate from both Upc and D2c (46% and 45% inhibition, respectively) (Figure 2; Table S2). In these two latter treatments, D1c samples caused lower yield reductions compared to D2c, yet this differential response among sites was not statistically significant. The toxic effect noted in sediments collected upstream of the effluent outfall are consistent with the higher PAH load of these sediment samples (approximately double that of D1c and D2c), while metal levels were similar across all sites within WWTPc (Table S1). This evidence suggests that the effluent is not a major source of metals or PAHs, even though WWTP effluents are often reported as significant sources of these contaminants [48,49]. The recipient waterway in WWTPc is urban, and therefore highly susceptible to diffuse contamination from traffic, which promotes the PAH input to waterbodies through runoff [50]. In the vicinity of UPc, traffic levels on a major road are notably higher compared to D1c and D2c. On the other hand, PPCPs load is higher in sediments downstream the effluent outfall, compared to Upc, which apparently does not agree with the toxicity trends. As previously discussed for other WWTP, the levels of PAH and metal contamination are several orders of magnitude bellow those that can cause impacts in *L. minor*. Responses of the microalgae to WWTPc samples exhibited high intra-sample variability, likely preventing the statistical confirmation of the growth impairment caused by elutriates from the three sites (Figure 3; Table S2). Although there seems to be a tendency for a reduction in growth following exposure to increasing elutriate concentrations regardless of the site, it should also be highlighted that the undiluted elutriates barely affected the microalgae growth, a pattern that is not consistent with what was observed for the macrophyte. Both species are model representatives of primary producers in ecotoxicological studies. However, their sensitivity to stressors often varies [51,52,53]; as the two species have similar metabolic pathways, thus potentially a similar toxicity mechanistic, constraints related to the different uptake routes available in each are likely a reason for the differential sensitivity observed herein. Indeed, microalgae lack a vascular system, which promotes surface contact as the single route by which contaminants can be uptake. Less lipophilic contaminants, for example, should be better uptake systemically (as in *L. minor*) than via surface contact (as in both species). Thus, it is possible that internal exposure to different contaminants in *L. minor* had been higher than in *R. subcapitata*, and hence the lower overall sensitivity of the microalgae to WWTPc elutriates.

Despite a clear relationship between the contaminants and the decreased growth of microalgae and macrophytes did not became evident in this study, our results should be held as indicative of the potential of WWTP effluents to induce noxious ecological effects. Photosynthetic producers are key to critical ecosystem functions, for example nutrient cycling, oxygen production or carbon sequestration, being as well drivers of energy and mass transfer to consumers through the food web [54,55].

### 3.3. Effects of Elutriates in Daphnia magna Reproductive Output

**Figure 4:**
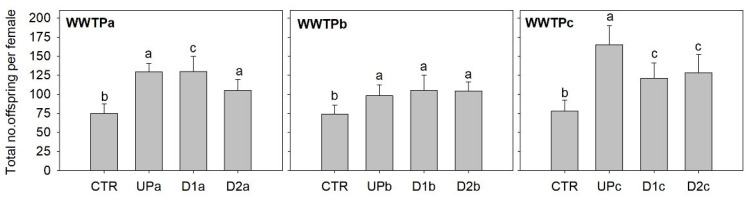
Total number of neonates per female *Daphnia magna*, released during the 21 days of exposure to sediment elutriates. The bars represent the average of ten replicates and the error bars represent the standard deviation. Letters denote differences among sites (Up, D1 and D2) and the control (CTR) within each WWTP (Tukey test; *p* < 0.05).

The reproduction of the waterflea *D. magna* was significantly stimulated rather than impaired by the elutriates of all samples, as clear from Figure 4 showing the total reproductive output per female following exposure to undiluted elutriates (statistics summary in Table S2). This stimulatory trend was observed in the other two parameters calculated after testing with *D. magna* (Tables S2 and S3): the first reproduction tended to become anticipated rather than delayed, significantly following exposure to samples from WWTPa and WWTPc; the size of the first brood increased significantly following exposure to samples from WWTPa and WWTPc. Sites within WWTPb resulted in similar reproductive output, while samples from downstream the effluent outfall tended to be less stimulatory to the daphnids reproduction than samples from upstream the effluent outfall in WWTPa and WWTPc (Figure 4; Table S2). Possible explanations for the stimulatory effects of elutriates in the reproductive outcome of *D. magna* include the mechanisms of action of some contaminants that are typically sourced by WWTP effluents, and/or the enrichment in organic matter provided by the elutriates compared to synthetic-media controls.

Selective serotonin reuptake inhibitors, used as antidepressants, have been claimed to induce increased offspring outputs in *D. magna* at low concentrations, while reducing reproductive output at high concentrations [56]. These pharmaceuticals are commonly detected in the environment and are often associated with WWTP effluents due to their recalcitrant behaviour towards commonly used wastewater treatment methods [57,58]. Silva et al. [18] quantified several antidepressants in all effluent samples and in the sediment samples downstream the effluent discharge of WWTPc, namely citalopram (11 and 8 µg/kg in D1c and D2c) amitriptyline (2.5 and 1.5 µg/kg in D1c and D2c) and sertraline (5 and 3 µg/kg in D1c and D2c) (Table S2), thus its availability for *Daphnia* intake during exposure can be expected. However, there is evidence that suspended sediments amplify the negative effects of citalopram on several biochemical and individual parameters assessed in *D. magna*, at concentrations several orders of magnitude higher than those potentially in place in the present study [59], but also at concentrations within the range or slightly above [60]. There is also evidence that sertraline impairs the heart rate and swimming velocity, as well as decreases the immediate reproductive output of *D. magna* at concentrations (0.1 and 1 µg/L) similar or below the range expected in our study [61]. Based on these literature reports and given that reproductive stimulation was found for all samples from all WWTPs and not just WWTPc, it is unlikely that antidepressants are the primary drivers of the effect noticed.

In the same line of reasoning, caffeine—quantified in sediments of WWTPc and in Upb (8.64–13.1 µg/kg and 0.20 µg/kg, respectively; Table S2)—is known to promote an anticipation of the first brood (as noticed herein; Table S2). Caffeine has also been shown to inhibit moulting and reduce offspring numbers at concentrations in the tenths of mg/L range [62]. However, at lower concentrations (up to 50 µg/L), caffeine can reduce daphnid movement without significantly affecting metabolic endpoints, such as oxygen uptake and glycogen reserves [63]. Alternatively, the stimulation in *D. magna* reproductive output could be driven by the organic enrichment prevalent in elutriates, which could provide some of the benefits commonly associated with supplements such as algae extract added for long-term *Daphnia* culturing (not added in the experiments; see Materials and Methods). In fact, organic matter from different sources can stimulate reproduction in daphnids and drive the offsetting of noxious effects of contaminants [64,65]. In WWTPa and WWTPb, the organic matter content in sediments is consistent with reproductive stimulation found (compare Figure 4 and Table S1): D2a sediments contain comparatively lower organic matter load, and the stimulatory effect was lower than that observed for Upa and D1a; for WWTPb, where all sediment samples had similar organic loads, the stimulatory effects on reproduction were also comparable across sites. However, this consistency does not extend to WWTPb: while Upc and D2c sediments bear similar organic load, Upc induced a significantly higher reproductive stimulation. In this latter case, it is possible that the higher contaminant concentrations found in sites downstream the effluent outfall (particularly PPCPs and some metals; see related discussions above) may have offset the stimulatory effect.

### 3.4. Effects of Elutriates in Chironomus riparius Growth

**Figure 5:**
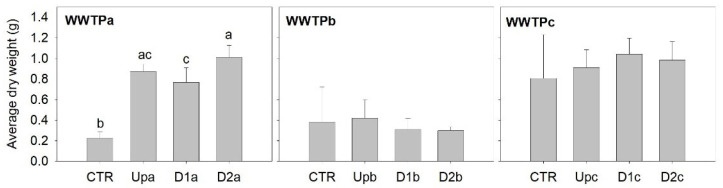
Average individual *Chironomus riparius* dry weight calculated after 28 days exposure to sediment samples. The bars represent the average of five replicates and the error bars represent the standard deviation. Letters denote differences among sites (Up, D1 and D2) and the control (CTR) within each WWTP (Tukey test; *p* < 0.05).

While for the previous model organisms, elutriate exposure was used to simulate resuspension conditions in which contaminants are mobilized into the water column [16] for *C. riparius* sediment samples were used directly, thereby covering another exposure route and increasing the realism of the test as whole sediment samples are the closest possible to the conditions in the field. The growth (on the basis of dry weight) of the midge *C. riparius* was significantly stimulated by the exposure to sediments collected in WWTPa, and unaffected by sediments collected in the other two WWTPs (Figure 5; Table S2). In WWTPa, there were no clear differences among sites and there was no clear distinction of differential stimulation between samples collected upstream and downstream the effluent outfall.

Consistently, other studies have already signalled the increase in midges’ dry weight after exposure to sediment collected in the vicinity of a small WWTP [66]. The organic matter supplementation provided by the tested natural sediment samples may have contributed to the stimulatory effects observed. As shown by Stuijfzand et al. [67], the success of *C. riparius* (often considered a “pollution-tolerant” midge) can be more likely explained by its ability to exploit organic matter enrichment of tested matrices as a superior food source than by the species argued tolerance to contaminants.

### 3.5. Insights on Consistency Between Ecotoxicological Responses and Ecological Quality

**Figure 6:**
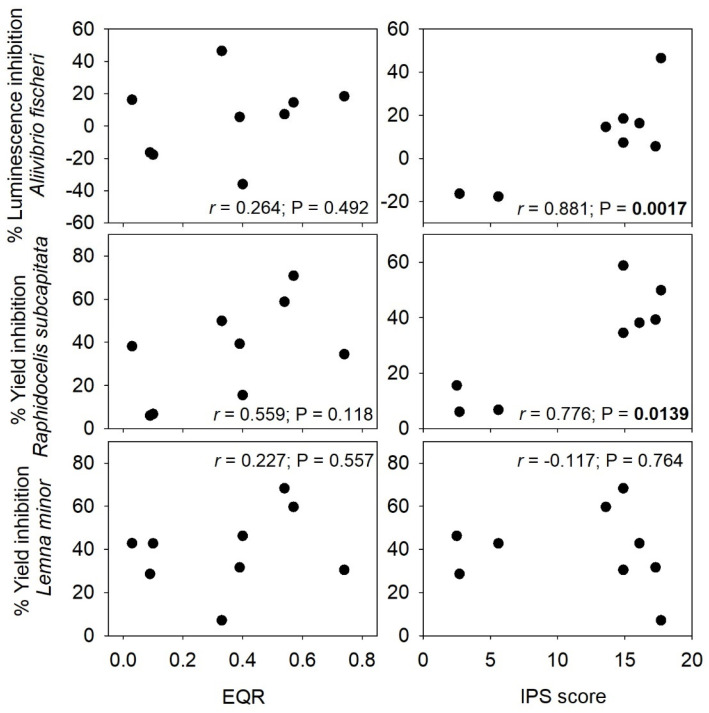
Correlations between endpoints negatively affected by sediment elutriates from all surveyed sites (the average percent inhibition on the endpoint compared to respective internal test controls for the undiluted elutriate treatment) and ecological quality determined for each site based on benthic macroinvertebrate communities (EQR—Ecological Quality Ratio; left-hand panel) and *Benthic diatom* communities (IPS score; right-hand panel) by Silva et al. [68]. The Pearson correlation coefficient (r) and associated significance are given for each corresponding plot; significant correlations are highlighted in bold.

The ecological quality of sites within each WWTP was previously assessed by Silva et al. [68], using two bioindicator communities as recommended for bioassessment under the European Water Framework Directive (WFD; Directive 2000/60/EC): benthic macroinvertebrates and periphytic diatoms [69]. This prior ecological assessment showed some inconsistencies between the ecological quality outcomes based on the two communities (see Table S4 for an immediate view of the ecological quality classifications of all sites). Despite this, we would expect that the ecotoxicological results herein obtained would be consistent with the picture assessed previously on the ecological quality statuses. Specifically, WWTPb sediments were expected to be the least toxic or even non-toxic, regardless of the model organism focused as both communities indicated towards moderate to high ecological quality. However, only animals were unaffected (actually the assessed endpoints were stimulated; Figure 4 and Figure 5) by WWTPb sediment samples. Animals were also unaffected by samples from WWTPa and WWTPc while the macroinvertebrates community indicated that sites within these WWTPs as bearing moderate (Upa) to poor (D1a, Upc) and bad (D2a, D1c, D2c). Conversely, diatoms suggested high to good ecological quality within WWTPa sites, even though sediment elutriates from these sites were toxic to bacteria, microalgae and macrophytes (Figure 1, Figure 2 and Figure 3). This unmatching between ecological and ecotoxicological assessments over the same sites within our three WWTP case studies is reinforced by correlation analysis ran to confirm the (dis)agreement between ecological quality standards as determined by Silva et al. [68] and the effects noticed in bacteria, microalgae and macrophytes (the analysis was logically not carried out for unaffected endpoints recorded in *D. magna* and *C. riparius*) after exposure to undiluted elutriates. The outcome of these correlations is shown in Figure 6, and it largely confirms the previously interpreted disagreement. No significant correlations were found between the endpoint records in toxicity tests and ecological quality ratios indicated by macroinvertebrate communities. Although correlations of bacteria luminescence records and microalgae yield with ecological quality scores as indicated by diatom communities have been found high and significant, these are positive correlations. This essentially means that toxicity (inhibition compared to internal test controls) increases as the ecological quality score increases, largely evidencing the unmatching between the two approaches to address the environmental hazardous potential of contaminated sites.

The analysis above supports the importance of integrating the ecotoxicological line of evidence as a complementary strategy in the standard bioassessment requested by major instruments such as the WFD. In fact, the use of effect-based tools such as bioassays within the context of ecological quality assessment has been claimed by several authors [69] and is actually a topic of intense reflexion at the EU level through specific working groups. For example, the European subgroup Chemical Monitoring and Emerging Pollutants under the Chemical Aspects working group of the Common Implementation Strategy for the WFD identified potential effect-based tools, including bioassays, that should be used in the WFD monitoring programmes as screening tools aiding in the prioritization of water bodies and sites; as a basis for early-warning systems; as indicators of effects of chemical mixtures or chemicals that are not monitored; and as a complementary support in water and sediment quality assessment [70].

Our results suggest that WWTP effluents affect the toxicity of sediments in recipient waterways, indicating negative or positive effects of exposure in different ecologically representative organisms. These effects hint on potential changes at different trophic levels, signalling potential disruption of ecosystem functioning and scaling-up concerns about the hazardous potential of WWTP discharge in the long term. Generally, regulatory environmental risk assessment frameworks and environmentally protective regulation addressing WWTP discharge rely on safety benchmarks for individual contaminants, based often in ecotoxicological assessment. Our results show that this approach is likely limited when complex matrices such as sediments receiving WWTP effluent inputs. The converse outcome of tests with different organisms is also worth noting. While a direct interpretation of the noxious potential of contaminated sediments can be made, the stimulatory responses by species such as daphnids and chironomids somehow bias the overall conclusions, consequently rendering it more challenging the communication of potential risks to regulators and managers. This picture is clear in demonstrating that the hazardous potential of WWTP effluents is far from being comprehensively understood so that feasible support can be given to decision makers and practitioners towards better environmental protection.

## 4. Conclusions

The present study was focused on the ecotoxicological effects of sediment samples collected in the vicinity of WWTPs, in the sequence of previous work characterizing the contaminants load of sediments in these same ecosystems. Our findings partially support the first initial hypothesis, demonstrating that sediment elutriates can indeed affect test organisms, although these impacts were generally mild. In certain cases, exposure to elutriates even appeared beneficial. This is the case for the *A. fischeri* test, where WWTPc elutriate samples caused a stimulatory effect. Similarly, both the *D. magna* and *C. riparius* tests showed stimulatory effects when exposed to elutriates or sediment samples compared to the control. In contrast, tests involving *L. minor* and *R. subcapitata* demonstrated significant negative impacts from exposure to elutriate samples. While not a primary objective of our study, these results highlight the importance of using a robust and diverse battery of test organisms; relying on a limited set of organisms can lead to skewed evaluations of sediment toxicity depending on whether the species used are overly sensitive or insensitive.

Regarding our second hypothesis on potential differences in ecotoxicological impacts between samples collected upstream and downstream effluent outfalls, although there is a slight tendency for increasingly deleterious effects in downstream samples, this raise was limited and not confirmed statistically. Thus, one cannot confidently claim that up and downstream sampling sites have differential hazardous potential.

There are important knowledge gaps regarding the toxic potential of WWTP originated effluents, whose complex compositions present challenges for predicting ecological impacts. Our work, planned for accommodating realistic assessments either using elutriates of real sediment samples or whole sediment samples, adds valuable insights to the field. Besides the immediate outcomes of the present ecotoxicological testing, discrepancies became apparent when compared to previous ecological assessment of the same samples, reinforcing that one approach is not necessarily predictive of the other. Although the testing approach was restricted to sediment samples, it is worth highlighting that the results are applicable to organisms inhabiting the water column provided that resuspension events are frequent, driven by natural processes such as bioturbation or storms or by anthropic actions such as dredging. Overall, the findings may inform future research and aid in decision-making related to environmental risk assessment and WWTP effluent management.

## Data Availability

The research data used in this article are presented with detail and information on data processing in the article or in the Supplementary Materials. Raw data are available upon reasonable request to the authors.

## Supplementary Materials

The following supporting information can be downloaded at: https://www.mdpi.com/article/10.3390/jox15040132/s1. Table S1: Summarized characterization of sampling sites (data collected from [18]); Table S2: Statistical summary regarding different one-way ANOVA; Table S3: Records on additional parameters calculated following the testing of elutriates from WWTP sediments with *Daphnia magna*; Table S4: Ecological status determined in all sites tested within WWTPa, WWTPb and WWTPc, using periphytic diatoms or macroinvertebrate communities, by Silva et al. [68].

## Abbreviations

The following abbreviations are used in this manuscript:

WWTPs	Wastewater treatment plant
CEC	Contaminants of emerging concern
PPCP	Pharmaceuticals and personal care products
PAHs	Polycyclic aromatic hydrocarbons
WFD	Water framework directive
OECD	Organisation for Economic Cooperation and Development
MBL	Woods Hole MBL medium
ASTM	American Society for Testing and Materials
