# Supplementary material for: Ecotoxicological Assessment of Sediment Samples Impacted by Wastewater Treatment Plant Effluents Transporting Contaminants of Emerging Concern
Source: J Xenobiot. 2025 Aug 15;15(4):132. doi: 10.3390/jox15040132 (PMC12387254; doi:10.3390/jox15040132)
Supplement: Supplementary file 1 [file jox-15-00132-s001.zip › jox-3741426-supplementary.pdf]

Ecotoxicological Assessment of Sediment Samples Impacted by Wastewater Treatment Plant Effluents Transporting Contaminants of Emerging Concern

Carlos Silva, Ana Ré, Nelson Abrantes, Fernando JM Gonçalves , Joana Luísa Pereira*

Supplementary material

Table S1. Summarized characterization of sampling sites (data collected from Silva et al., 2022). General water and sediment quality parameters are provided first, then quantified concentrations (dry weight) of PPCPs, metallic elements, and PAHs in sediments are given for compounds found in at least one sample analyzed in the present study.

	WWTPa			WWTPb			WWTPc		
	Upa	D1a	D2a	Upb	D1b	D2b	Upc	D1c	D2c
Water									
Flow speed (m/s)	11.11	12.36	0.25	25	27.22	9.72	7.69	9.64	8.99
Temperature (ºC)	18.3	17.7	17.2	17.3	18.2	17.8	28.9	24.1	23.6
Conductivity (µS/cm)	398	400	360	505	458	1435	619	799	1025
Dissolved oxygen (mg/L)	8.81	8.94	7.85	9.88	9.46	9.72	12.26	5.30	5.42
Oxygen saturation (%)	93.9	93.7	81.1	102	100.3	102.6	158.7	63.4	63.8
pH	7.13	7.35	7.37	7.24	7.41	7.03	7.64	8.45	8.28
Total dissolved solids, TDS (mg/L)	256	260	ND	328	297	933	402	521	ND
Mean water depth (cm)	41	60	37	50	40	50	20	20	60
Total Nitrogen, N (mg/L)	0.28	0.00	0.00	0.00	0.00	17.41	19.32	34.56	30.59
Total Phosphorous, P (mg/L)	0.00	0.03	0.00	0.00	0.07	0.05	0.17	0.49	0.64
Sediment									
Organic matter (% w/w)	1.34	1.34	0.19	0.44	0.37	0.44	0.49	0.58	0.42
Silt >3.9-63 µm (% w/w)	4.67	4.67	0.43	1.21	3.49	6.41	2.47	2.72	3.46
Sand >63-2000 µm (% w/w)	95.12	95.12	94.86	83.06	90.79	92.40	90.01	76.02	86.62
Gravel >2000 (% w/w)	0.23	0.23	4.70	15.72	5.71	1.20	7.53	21.27	9.91
WWTP characteristics									
Population equivalents served	160,000			50,000			700,000		
Primary treatment	settling and equalization			settling and equalization			settling and equalization		
Secondary treatment	None			Biological reactor			Biological reactor, settling in and biofiltration		
PPCPs (µg/kg)									
Amisulpride	bdl	bdl	bdl	bdl	bdl	bdl	bql	1.52	1.13
Amitriptyline	bdl	bdl	bdl	bdl	bdl	bdl	bdl	2.48	1.54
Atenolol	bdl	bdl	bdl	bdl	bdl	bdl	bdl	1.19	1.13
Bisoprolol	bdl	bdl	bdl	bdl	bdl	bdl	bdl	1.19	1.21
Caffeine	bql	bql	bql	bql	bql	0.20	13.1	15.9	8.64
Citalopram	bdl	bdl	bdl	bdl	bql	bql	bql	10.95	7.67
Propranolol	bdl	bdl	bdl	bdl	bdl	bdl	bdl	3.14	3.09
Sertraline	bdl	bdl	bdl	bdl	bdl	bdl	bdl	4.58	3.41
Tramadol	bdl	bdl	bdl	bdl	bdl	bdl	bql	20.2	22.5
Triclosan	bdl	bdl	bdl	bdl	bdl	bdl	bdl	1.72	1.35
Trimethoprim	bdl	bdl	bdl	0.07	48.0	bql	bql	bql	bql
Venlafaxine	bdl	bdl	bdl	bql	bql	bdl	bql	12.4	10.1
Metals and metalloids (mg/Kg)									
Li	1.91	1.38	0.62	1.17	1.51	1.32	2.98	3.38	3.33
Be	0.18	bdl	bdl	bdl	bdl	bdl	0.29	0.52	bdl

Na	bdl	bdl	bdl	bdl	bdl	bdl	294	321	220
Mg	174	105	35.0	292	365	343	4389	5012	4804
Al	1341	790	303	662	1005	984	5107	6221	5508
P	172	253	94.7	101	116	118	651	685	827
K	304	212	bdl	bdl	207	238	585	745	687
Ca	1122	796	185	36393	35934	22553	40874	40602	38754
V	1.54	0.85	0.33	1.85	2.29	2.35	23.0	26.6	24.1
Cr	1.71	1.07	bdl	1.92	2.94	2.65	17.7	23.7	22.2
Mn	21.8	14.4	4.71	52.3	33.7	49.5	210	192	182
Fe	2762	1452	660	1631	2092	2225	11237	12232	11466
Co	0.64	bdl	bdl	1.93	1.48	1.66	8.53	10.6	10.65
Ni	1.51	1.02	bdl	1.74	2.23	2.28	21.6	26.0	25.15
Cu	3.92	4.62	0.73	3.72	4.25	5.00	11.06	19.3	20.88
Zn	11.9	9.63	2.54	9.05	15.4	15.9	50.2	84.8	102.75
As	0.88	bdl	bdl	bdl	bdl	bdl	0.78	0.71	0.81
Se	bdl	bdl	bdl	bdl	bdl	bdl	0.63	0.70	0.67
Rb	3.30	1.97	0.95	1.77	2.85	2.86	3.98	5.04	4.92
Sr	3.89	3.53	2.04	19.9	43.4	14.7	72.1	76.9	68.1
Y	1.20	0.62	0.32	1.20	1.31	1.38	3.43	3.77	3.74
Mo	bdl	bdl	bdl	bdl	bdl	bdl	0.22	1.34	0.25
Ag	0.05	bdl	bdl	bdl	bdl	0.34	0.23	0.23	0.66
Cd	bdl	bdl	bdl	bdl	bdl	bdl	0.09	0.09	0.96
In	bdl	bdl	bdl	bdl	bdl	bdl	bdl	bdl	bdl
Sn	0.22	0.25	0.53	0.23	0.22	0.22	0.13	0.50	0.61
Sb	bdl	bdl	bdl	bdl	bdl	bdl	bdl	bdl	bdl
Ba	13.7	10.7	30.3	11.6	834	15.0	66.1	61.4	64.0
La	3.06	1.69	1.15	2.67	3.53	4.73	9.85	13.6	10.0
Ce	6.47	3.54	2.39	5.54	7.32	10.3	19.3	21.4	20.2
Pr	0.80	0.43	0.29	0.68	0.88	1.20	2.38	2.61	2.45
Nd	3.12	1.70	1.08	2.63	3.43	4.53	9.37	10.4	9.81
Sm	0.58	0.31	0.21	0.53	0.77	0.83	1.69	1.86	1.74
Eu	0.12	0.08	0.12	0.11	2.97	0.16	0.64	0.66	0.65
Gd	1.12	0.61	0.41	0.96	1.44	1.72	3.38	3.67	3.49
Tb	0.09	0.05	0.03	0.08	0.10	0.13	0.28	0.31	0.30
Dy	0.31	0.16	0.10	0.28	0.33	0.40	0.98	1.07	1.05
Ho	0.05	0.03	0.01	0.05	0.05	0.06	0.18	0.19	0.18
Er	0.13	0.07	0.03	0.11	0.13	0.14	0.41	0.44	0.43
Tm	0.02	0.01	0.01	0.02	0.02	0.02	0.07	0.07	0.06
Yb	0.09	0.04	0.01	0.08	0.08	0.10	0.28	0.30	0.29
Lu	bdl	bdl	bdl	bdl	bdl	bdl	0.05	0.04	0.04
Pb	2.08	1.32	1.09	2.67	56.38	4.98	10.28	6.32	8.08
U	0.17	0.08	0.09	0.23	0.27	0.31	0.34	0.35	0.31
PAHs (µg/Kg)									
Naphthalene (NP)	bdl	bdl	bdl	2.49	2.49	3.73	1.71	2.05	2.91
Acenaphthylene (ACY)	bdl	bdl	bdl	bdl	bdl	bdl	bdl	1.26	1.69
Acenaphthene (ACE)	bdl	bdl	bdl	bdl	1.77	bdl	3.86	bdl	bdl
Fluorene (FLU)	bdl	bdl	bdl	bdl	bdl	bdl	6.66	6.13	bdl
Phenanthrene (PHE)	1.85	2.01	1.35	1.61	1.01	1.22	32.70	24.30	11.90
Anthracene (ANT)	bdl	bdl	bdl	bdl	bdl	bdl	5.90	bdl	bdl
Fluoranthene (FLA)	1.76	2.21	1.26	1.99	1.15	1.59	44.10	28.60	17.70
Pyrene (PYR)	3.25	3.87	2.12	2.25	1.17	1.64	48.40	52.10	25.40
Benzo[a]anthracene (BAA)	bdl	bdl	bdl	1.34	bdl	bdl	16.30	1.71	4.14
Chrysene (CRY)	bdl	bdl	bdl	2.15	1.52	1.73	16.30	3.42	5.50
Benzo[b]fluoranthene (BBF)	bdl	bdl	bdl	1.60	1.00	1.33	16.30	2.87	7.04
Benzo[k]fluoranthene (BKF)	bdl	bdl	bdl	bdl	bdl	bdl	8.19	1.64	3.50
Benzo[a]pyrene (BAP)	bdl	bdl	bdl	1.17	bdl	1.22	23.80	2.81	8.62
Indeno[1,2,3-c,d]pyrene (IND)	bdl	bdl	bdl	bdl	bdl	bdl	15.80	2.49	7.16
Dibenzo[a,h]anthracene (DBAH)	bdl	bdl	bdl	bdl	bdl	bdl	3.53	bdl	bdl
Benzo[g,h,i]perylene (BGHI)	bdl	bdl	bdl	1.46	1.26	1.78	18.90	3.41	9.63
PAHs sum	6.86	8.10	4.73	16.06	11.37	14.24	262.45	132.79	105.19

bdl – below detection limit; bql – below quantification limit; ND – Not determined (TDS was not determined for WWTPc D2 due to technical problems with the probe in the field). Sediment size classification according to Wentworth, C. K., 1922. A Scale of Grade and Class Terms for Clastic Sediments Author (s): Chester K. Wentworth Published by: The University of Chicago Press

Table S2 Statistical summary regarding different one-way ANOVA. The F value or the H value, along with degrees of freedom and associated P value, are presented depending on the previous confirmation of ANOVA assumptions normality and homoscedasticity (parametric F test) or failure of one of the assumptions (Kruskal-Wallis H test).

**Organism**	**Parameter**	**WWTP**	**Effect tested/endpoint**	**Statistical analysis summary**
Lemna minor	Yield (number of fronds)	A	Upa elutriate dilution	H_2 = 12.222; p = **0.032**
			D1a elutriate dilution	$F_{5, 15}$ = 1.759; p = 0.182
			D2a elutriate dilution	$F_{5, 15}$ = 8.789; p **<0.001**
			75% elutriate between sites	$F_{2, 6}$ = 9.924; p = **0.013**
			100% elutriate between sites	$F_{2, 6}$ = 4.711; p = 0.059
		B	Upb elutriate dilution	$F_{5, 15}$ = 14.068; p **<0.001**
			D1b elutriate dilution	$F_{5, 15}$ = 17.983; p **<0.001**
			D2b elutriate dilution	$F_{5, 15}$ = 4.258; p = **0.013**
			50% elutriate between sites	$F_{2, 6}$ = 1.485; p = 0.299
			75% elutriate between sites	$F_{2, 6}$ = 6.925; p = **0.028**
			100% elutriate between sites	$F_{2, 6}$ = 23.471; p = **0.001**
		C	Upc elutriate dilution	$F_{5, 15}$ = 6.945; p = **0.002**
			D1c elutriate dilution	$F_{5, 15}$ = 3.894; p = **0.018**
			D2c elutriate dilution	$F_{5, 15}$ = 6.974; p = **0.001**
			12.5% elutriate between sites	$F_{2, 6}$ = 5.697; p = **0.041**
			75% elutriate between sites	H_2 = 5.468; p = 0.071
			100% elutriate between sites	$F_{2, 6}$ = 2.168; p = 0.196
Raphidocelis subcapitata	Yield (cell density)	A	Upa elutriate dilution	$F_{5, 27}$ = 3.143; p = **0.023**
			D1a elutriate dilution	$F_{5, 27}$ = 5.952; p **<0.001**
			D2a elutriate dilution	$F_{5, 27}$ = 6.866; p **<0.001**
			12.5% elutriate between sites	$F_{2, 6}$ = 3.387; p = 0.104
			50% elutriate between sites	$F_{2, 6}$ = 7.250; p = **0.025**
			75% elutriate between sites	$F_{2, 6}$ = 1.561; p = 0.285
			100% elutriate between sites	$F_{2, 6}$ = 0279; p = 0.766
		B	Upb elutriate dilution	$F_{5, 27}$ = 11.907; p **<0.001**
			D1b elutriate dilution	$F_{5, 27}$ = 17.399; p **<0.001**
			D2b elutriate dilution	H_5 = 14.814; p = **0.011**
			12.5% elutriate between sites	$F_{2, 6}$ = 2.960; p = 0.128
			25% elutriate between sites	$F_{2, 6}$ = 7.332; p = **0.024**
			75% elutriate between sites	$F_{2, 6}$ = 3.557; p = 0.096
			100% elutriate between sites	$F_{2, 6}$ = 3.154; p = 0.116
		C	Upc elutriate dilution	H_5 = 9.360; p = 0.096
			D1c elutriate dilution	H_5 = 14.367; p = **0.013**
			D2c elutriate dilution	H_5 = 11.032; p = 0.051
			75% elutriate between sites	$F_{2, 6}$ = 3.134; p = 0.107
Daphnia magna		A	Age at First Reproduction	H_3 = 23.201; p **<0.001**
			Size of first brood	H_3 = 15.654; p = **0.001**
			Total no. neonates	$F_{3, 36}$ = 31.420; p **<0.001**
		B	Age at First Reproduction	$F_{3, 35}$ = 2.403; p = 0.084
			Size of first brood	$F_{3, 35}$ = 0.240; p = 0.868
			Total no. neonates	$F_{3, 35}$ = 9.579; p **<0.001**
		C	Age at First Reproduction	H_3 = 15.581; p = **0.001**
			Size of first brood	$F_{3, 35}$ = 17.081; p **<0.001**
			Total no. neonates	$F_{3, 34}$ = 27.315; p **<0.001**
Chironomus riparius		A	Average dry weight	$F_{3, 16}$ = 55.375; **p <0.001**
		B	Average dry weight	$F_{3, 16}$ = 0.430; p =0.734
		C	Average dry weight	$F_{3, 12}$ = 0.774; p =0.530

Table S3 Records on additional parameters calculated following the testing of elutriates from WWTP sediments with *Daphnia magna*. Values presented are the arithmetic mean of 10 replicates, with standard deviation presented within brackets.

	Sample	Age at First Reproduction (days)	Size of the first brood
**WWTPa**	**CTR**	10.30 (1.03)	7.75 (3.53)
	**Upa**	9.30 (0.48)	9.01 (2.07)
	**D1a**	9.00 (0.00)	12.63 (2.64)
	**D2a**	11.00 (2.12)	14.11 (9.81)
**WWTPb**	**CTR**	10.30 (0.68)	12.80 (3.77)
	**Upb**	9.70 (0.68)	11.50 (3.47)
	**D1b**	10.10 (0.32)	12.60 (3.89)
	**D2b**	10.00 (0.00)	13.00 (4.72)
**WWTPc**	**CTR**	9.80 (0.42)	8.50 (3.03)
	**Upc**	8.80 (0.42)	18.30 (3.16)
	**D1c**	9.25 (0.46)	13.78 (1.86)
	**D2c**	8.80 (0.92)	20.30 (6.34)

Table S4. Ecological status determined in all sites tested within WWTPa, WWTPb and WWTPc, using periphytic diatoms or macroinvertebrate communities, by Silva et al. (2024).

Indicator group	Sample	Ecological status
**Macroinvertebrates**	Upa	Moderate
	D1a	Poor
	D2a	Bad
	Upb	Moderate
	D1b	Good
	D2b	High
	Upc	Poor
	D1c	Bad
	D2c	Bad
**Diatoms**	Upa	High
	D1a	High
	D2a	Good
	Upb	Good
	D1b	Good
	D2b	Good
	Upc	Bad
	D1c	Bad
	D2c	Poor

References

Silva, C.; Cachada, A.; Gonçalves, F.J.M.; Nannou, C.; Lambropoulou, D.; Patinha, C.; Abrantes, N.; Pereira, J.L. Chemical Characterization of Riverine Sediments Affected by Wastewater Treatment Plant Effluent Discharge. *Sci. Total Environ.* **2022**, *839*, 156305.

Silva, C.; Santos, J.I.; Vidal, T.; Silva, S.; Almeida, S.F.P.; Gonçalves, F.J.M.; Abrantes, N.; Pereira, J.L. Potential Effects of the Discharge of Wastewater Treatment Plant (WWTP) Effluents in Benthic Communities: Evidence from Three Distinct WWTP Systems. *Environ. Sci. Pollut. Res.* **2024**, *31*, 34492–34506
